# Exploring White Matter Microstructure with Symptom Severity and Outcomes Following Deep Brain Stimulation in Tremor Syndromes

**DOI:** 10.5334/tohm.904

**Published:** 2024-08-28

**Authors:** Luke Andrews, Simon Keller, Corey Ratcliffe, Jibril Osman-Farah, Hilary Shepherd, Maneesh Bhojak, Antonella Macerollo

**Affiliations:** 1 Institute of Systems, Molecular and Integrative Biology, University of Liverpool, UK; 2 The Walton Centre NHS Foundation Trust for Neurology and Neurosurgery, Liverpool, UK

**Keywords:** essential tremor, dystonic tremor, fixel-based analysis, deep brain stimulation

## Abstract

**Background:**

Essential tremor (ET) and dystonic tremor (DT) are movement disorders that cause debilitating symptoms, significantly impacting daily activities and quality of life. A poor understanding of their pathophysiology, as well as the mediators of clinical outcomes following deep brain stimulation (DBS), highlights the need for biomarkers to accurately characterise and optimally treat patients.

**Objectives:**

We assessed the white matter microstructure of pathways implicated in the pathophysiology and therapeutic intervention in a retrospective cohort of patients with DT (n = 17) and ET (n = 19). We aimed to identity associations between white matter microstructure, upper limb tremor severity, and tremor improvement following DBS.

**Methods:**

A fixel-based analysis pipeline was implemented to investigate white matter microstructural metrics in the whole brain, cerebello-thalamic pathways and tracts connected to stimulation volumes following DBS. Associations with preoperative and postoperative severity were analysed within each disorder group and across combined disorder groups.

**Results:**

DBS led to significant improvements in both groups. No group differences in stimulation positions were identified. When white matter microstructural data was aligned according to the maximally affected upper limb, increased fiber density, and combined fiber density & cross-section of fixels in the left cerebellum were associated with greater tremor severity across DT and ET patients. White matter microstructure did not show associations with postoperative changes in cerebello-thalamic pathways, or tracts connected to stimulation volumes.

**Discussion:**

Diffusion changes of the cerebellum are associated with the severity of upper limb tremor and appear to overlap in essential or dystonic tremor disorders.

## Introduction

Essential tremor (ET) and dystonic tremor (DT) are neurological conditions characterized by involuntary rhythmic movements, typically affecting the hands, arms, or head. Essential tremor is among the most common movement disorders, involving rhythmic shaking typically occurring during voluntary movements [1]. Dystonic tremor typically features an irregular tremor associated with specific tasks or postures, and occurs in a body area affected by dystonia [2]. The clinical overlap between these tremor disorders can complicate diagnosis, especially if dystonic signs are soft or are not the primary complaint [3,4]. Additionally, the term ET plus has been introduced to describe cases of essential tremor with non-typically presenting signs, such as resting tremor [2]. The pathophysiological understanding of these disorders is currently lacking, highlighting the need for further research.

A similar tremor oscillator has been theorised to underpin essential and dystonic tremor syndromes [5], and research has frequently implicated the cerebellum and thalamus, although their exact roles remain unclear [6,7,8]. Alterations in cerebello-thalamic connectivity, and in particular, the dentato-rubro-thalamic tract, has been proposed as a pathophysiological mechanism [9,10,11], with stimulation of these tracts using deep brain stimulation (DBS) ameliorating tremor in ET [12,13], as well as DT [14]. A weakened cerebello-thalamic connection has been specifically proposed to result in instability of the motor network, giving rise to tremor in DT [15]. Alterations to the integrity of cerebello-thalamic connections may contribute to variability in symptom severity across tremor syndromes, as well as mediating postoperative DBS improvements, even if sufficient targeting accuracy is achieved.

Diffusion weighted imaging (DWI) offers a means to assess water diffusion, and therefore, derive proxy measures of change in white matter microstructure [16]. Fixel-based analysis (FBA) is an analytic framework for DWI [17,18], that takes a voxel-based fiber-centric approach, abridged to “fixel”. FBA may provide inference into the morphometry of individual pathways, and therefore, has been proposed to overcome limitations of diffusion tensor models which are unable to model multiple fiber orientations that are ubiquitous throughout the brain [19,20]. FBA has been widely applied to identify alterations across many conditions and disorders. Examples in movement disorders include Parkinson’s disease [21], cervical dystonia [22], and Huntington’s disease [23]. Preoperative DWI sequences may be used to assess metrics of white matter microstructure in relation to both pre-and postoperative outcomes [24,25].

In the present exploratory study, we hypothesized that tremor severity and the potential for DBS to mediate tremor may be quantified within the microstructure of preoperative white matter fixel metrics. This hypothesis was tested in a retrospective clinical cohort of patients with ET (and ET plus) and DT who were treated with DBS at our center. Aside from exploratory whole-brain analyses, we also focused particularly on the cerebello-thalamic substrate, testing for associations of fiber metrics with preoperative tremor severity and the possibility for tremor mediation following DBS. Moreover, we aimed to test for microstructural associations of white matter fixel metrics in tracts connected to stimulation volumes across the cohort in relation to postoperative change.

## Methods

### Patient Selection

The data from a retrospective cohort of 38 patients (17 patients with DT and 21 patients with ET; 13 patients diagnosed as ET plus) treated at the Walton Centre NHS Foundation Trust, Liverpool, UK between 2013 and 2022 were initially included for the present study. Ethical approval for this study was granted by a local ethical committee (Health Research and Care Wales; reference number: 22/PR/1326).

All patients met the following criteria: (1) a confirmed diagnosis of pharmaco-resistant ET characterised by postural and intentional tremor components, ET plus with an additional resting tremor component and no presence of dystonic soft signs or parkinsonism (confirmed by a negative DaTSCAN in suspect cases) or DT whose primary complaint was severe tremor (not tremor associated with dystonia), (2) no history of stereotactic brain surgery, (3) no signs or history of other neurological disease or disorder, (4) availability of pre-and-postoperative clinical and demographic information, and (5) availability of preoperative DWI.

### Surgical Targeting and Clinical Outcomes

MRI were obtained, and surgical planning was performed 24 hours prior to implantation, using Framelink Medtronic between 2012–2018 and Elements BrainLab from 2018 to the current date. Automated segmentation of basal ganglia nuclei (posterior subthalamic area (PSA), zona incerta (ZI) and ventral intermediate nucleus (VIM)) was performed on the preoperative MRI using Elements Segmentation (version 5.0.0.72, Brainlab AG, Munich, Germany) and was systematically reviewed by the neurosurgeon (JOF). Targets were planned for the border of the VIM and ventralis oralis posterior (VOp). Intraoperative microelectrode recordings following macrostimulation were used to adjust lead placement to determine optimal locations for tremor reduction.

Following 2019, preoperative diffusion-weighted MRI was used to inform placement postoperatively but did not guide targeting intraoperatively. All patients were implanted with bilateral eight-contact Vercise™ Boston scientific DBS leads with the latest available pulse generators (Vercise IPG/Gevia IPG Boston Scientific).

The Fahn Tolosa Marin Tremor Rating Scale (FTMTRS) was used to quantify the severity of tremor. Right-sided items 5, 11, 12 and 13, and left-sided items 6, 11, 12 and 13 were used to evaluate lateralised upper limb improvements. Raw scores were utilised to quantify preoperative upper limb tremor severity. To measure post-DBS upper limb improvements, a percent change score was calculated using the following formula: \[\left({{\textstyle{{PR{E_{LAT}} - POS{T_{LAT}}} \over {PR{E_{LAT}}}}}} \right)*100\]. A short-term (< 2-years) postoperative follow-up time point was utilised given the recency of implantations, and lack of subsequent long-term follow-up data across the cohort.

### MRI acquisition

Due to the clinical and retrospective nature of the study, data from the cohort consisted of two different scanning protocols acquired from the same scanner (Siemens 3T Skyra; 20-channel head coil). In 21 patients (11 patients with DT; 5 patients with ET plus), diffusion acquisition consisted of 60 directions at *b* = 1,000 s/mm^–2^ with eight *b* = 0 s/mm^–2^ volumes acquired at 1.88 × 1.88 × 3 voxel resolution (TR/TE = 7.2/0.089 seconds, respectively). In 17 patients (6 patients with DT; 6 patients with ET plus), diffusion acquisition consisted of 30 directions at *b* = 1000 s/mm^–2^ with one *b* = 0 s/mm^–2^ volume acquired at 1.72 × 1.72 × 2 voxel resolution (TR/TE = 12.5/0.08 seconds, respectively).

### Diffusion pre-processing

Briefly, diffusion data were denoised (*dwidenoise*) and Gibbs ringing artefacts were removed (*degibbs*) using commands from the MRtrix3 software package [26]. As no opposite phase-encoded b0 volume was acquired, Synb0-DISCO was used to synthesise a reverse phase-encoded image. [https://gihub.com/MASILab/Synb0-DISCO; [27]] The FMRIB software library (FSL) (FMRIB, Oxford, UK) was then used to correct for susceptibility induced distortions and head motion (using topup; [28]) and eddy current distortion (using eddy; [29]). DWI data was brain extracted using SynthStrip, a contrast agnostic approach for brain extraction [30].

### Electrode Modelling and Stimulation Reconstruction

Lead DBS (version 3.0; https://www.lead-dbs.org) was used for electrode modelling and reconstruction [31]. Preoperative T1-weighted magnetisation-prepared rapid gradient-echo (MPRAGE) and T2-weighted or FLAIR images were co-registered using SPM [32]. Preoperative MRI were co-registered with CT images using a two-stage linear registration as implemented in Advanced Normalisation Tools (ANTs; http://stnava.github.io/ANTs/; [33]). Imaging sequences were spatially normalised to a non-linear adult template space (MNI 2009b nonlinear asymmetric space; [34]) by applying the ANTs SyN Diffeomorphic mapping with the “low variance + subcortical refinement” preset [35]. Precise nonlinear deformation was achieved in five stages: After two linear (rigid followed by affine) steps, a whole brain non-linear SyN-registration stage was followed by two nonlinear low-variance SyN-registrations focused on subcortical refinement using a coarse mask [36]. Perioperative pneumocephalus induced brainshift in postoperative acquisitions was corrected for by applying a refined affine transformation calculated between pre- and postoperative acquisitions that were restricted to a subcortical area of interest [37]. WarpDrive was then used to refine the fit of the thalamus, using available T1 and T2 or FLAIR acquisitions per patient [38].

Lead trajectories were automatically determined using the PaCeR algorithim [39], and were manually refined according to artifacts present in the postoperative CT image. DBS potential distribution volumes were simulated using the SimBio/FieldTrip pipeline [40]. Normalised scenes were checked for visual corroboration with native space scenes using artefacts from overlaid co-registered preoperative MRI and postoperative CT images.

### Fixel processing

**Figure 1:**
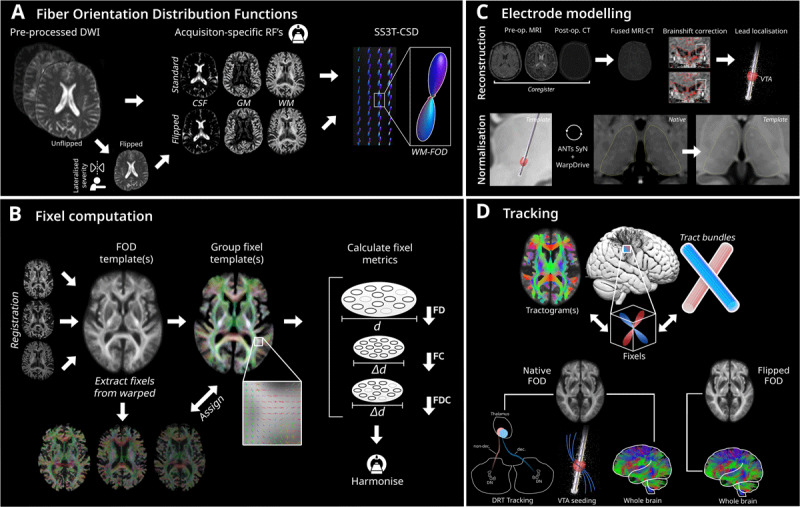
**Study pipeline. A)** DWI were pre-processed and then their native and flipped orientations (contralateral to the upper limb with highest preoperative tremor severity) were used to compute response functions separately for each acquisition scheme for the basis of single-shell 3-tissue constrained spherical deconvolution. **B)** Native and flipped white matter FOD images were used to compute study-specific templates. Fixel metrics (FD, FC (Δ*d* indicating change in diameter) and FDC) were computed and harmonised according to acquisition scheme using neuroCombat. **C)** DBS stimulation volumes were reconstructed for each patient. **D)** Tractograms were generated from each white-matter FOD template. Dentato-rubro-thalamic tracts were reconstructed to extract FDC metrics. Whole-brain and stimulation-seeded general linear models were used to model associations of preoperative tremor severity and postoperative tremor change, respectively. Abbreviations: dentato-rubro-thalamic, DRT (decussating, dec.; non-decussating, non-dec.); deep brain stimulation, DBS; cerebrospinal fluid, CSF; diffusion-weighted imaging, DWI; fiber cross-section, FC; fiber density, FD; fiber density and cross-section, FDC; fiber orientation distribution, FOD; grey matter, GM; primary motor cortex, M1; response function, RF; volume of tissue activation, VTA; white matter, WM.

The pipeline used for the present study is outlined in Figure 1. FBA was implemented in MRtrix3 and commands from a forked version of the MRtrix3 software package, MRtrix3Tissue [https://3Tissue.github.io]. Data were first bias field corrected (*dwibiascorrect*). Average response functions for white matter, grey matter and cerebrospinal fluid were computed for each patient, grouped by their diffusion acquisition scheme to account for differences in protocols (*dwi2response dhollander*) from the data using an unsupervised method [41]. Fiber orientation distribution (FOD) functions were computed from the three tissues using single shell constrained spherical deconvolution (*SS3T-CSD*) [42]. FODs for each tissue were bias field corrected and the global intensity was normalised (*mtnormalise*). An FOD template was created using all patient data, and warps were computed to register each patient to the templates. Subsequently, a fixel mask was generated from the FOD template.

Although tremor presentation was bilateral in this cohort, more subtle alterations to white matter microstructure may be present on the less dominant hemisphere, and thus, detectability through diffusion metrics may be obscured. To address this, we employed a separate severity-based lateralisation approach. A laterality index (LI) was used to identify the more severely affected upper limb and was calculated using the formula: \[PR{E_{RIGHT}}-PR{E_{LEFT}}\]. Fewer patients presented with a more affected left upper limb tremor (negative LI). Pre-processed diffusion data were flipped across the x-axis to align more severely impacted hemispheres across patients (contralateral to the upper limb), and therefore, potentially enhance the detection of microstructural white matter changes associated with tremor severity. Patients with right-lateralised upper limb severity (positive LI) or equal severity across hemispheres (zero LI) were left in native orientation. The data were then re-processed following the steps in the paragraph above.

Tractograms for the native and flipped pipelines were generated using whole-brain, second-order fiber orientation distribution probabilistic tractography [43]. Twenty million streamlines were generated using the recommended tracking angle (22.5°) and lengths (min 10; max 250) [20], followed by applying the spherical-deconvolution informed filtering of tractograms (SIFT) algorithm (maintaining 2 million streamlines) to reduce reconstruction biases [44]. Fixel-fixel connectivity—calculated as the shared number of fixels between two fixels relative to all the streamlines associated with the fixel being estimated from— matrices were generated and smoothed.

Fixel metrics were harmonised to account for differences in acquisition scheme using an implementation of neuroCombat within the fixel pipeline [45,46]. Given a lack of current imaging evidence and consensus to support ET and ET plus as entirely distinct disorders, we chose to combine patients into a single group. Harmonisation was performed using preoperative tremor severity (left and right separately for the native analysis and the maximally affected lateralised score for the flipped analyses), with age, sex and disorder group (ET or DT) as additional covariates. Intracranial volume was further included for the (log-scaled) fiber cross-section (FC_log_) and combined fiber-density and cross-section (FDC) metrics due to the potential association of head size and fiber cross-section (and the subsequent calculation of fiber cross-section with fiber density (FD)) [47]. SynthStrip [30] was again used to brain extract contrast-enhanced T1-weighted MRI (acquired as part of surgical planning). Intracranial volume was calculated by multiplying the brain extracted mask by the voxel spacing.

### Stimulation seeded analyses

Patient left and right hemispheric volume of tissue activated (VTA) were transformed into the native template space using MNI to template warps. VTA here were defined as e-field magnitude vectors binarised at ≥ 0.2 V/mm, as biophysical models have inferred this heuristic as a threshold value to invoke axonal stimulation [48]. Each patient VTA was used to seed streamlines from the SIFT tractogram (*tckedit*). Tracks were then transformed into voxel maps (*tck2fixel* & *fixel2voxel*) and aggregated to create disorder, and hemispheric-specific *N*-maps (where larger values indicate visitation of voxels over a greater number of patients). The lowest 10% of visited voxels were excluded from each *N*-map to remove unfrequented regions.

### Dentato-rubro-thalamic tracts

Left and right non-decussating and decussating dentato-rubro-thalamic pathways were defined according to known anatomical trajectories [49]. Left and right dentate nucleus (seed regions), superior cerebellar peduncle (waypoint region), red nucleus (waypoint region) and thalamus (stop region) were manually defined on the native FOD template and confirmed for accuracy by a trained anatomist.

Decussating cerebello-thalamic pathways were tracked as sequential inclusions for the ipsilateral dentate nucleus, ipsilateral superior cerebellar peduncle, contralateral red nucleus and contralateral thalamus. Non-decussating cerebello-thalamic pathways were tracked as ordered inclusions for the ipsilateral dentate nucleus, ipsilateral superior cerebellar peduncle, ipsilateral red nucleus and ipsilateral thalamus. For each tract, 2000 streamlines were generated (*tckgen*), followed by applying SIFT to maintain 200 streamlines. Note, this value was arbitrary but was used to ensure a sufficient number of fixels were present in each pathway.

### Demographic and clinical variables

For demographic and clinical outcome data, assumptions of normality were tested using Shapiro-Wilk tests. Wilcoxon-signed rank tests, or paired *t* tests were used to assess improvements in groups following DBS. Wilcoxon rank sum or unpaired *t* tests were used to compare between groups. Differences in sex were tested for using χ^2^. Pearson’s (*R*_p_) or Spearman’s rank (*R*_s_) correlations were used to assess associations of age and disease duration with clinical outcomes between groups, where appropriate. Statistical significance was set at *p* < 0.05. Analyses were performed using R (version 4.3.1).

### Whole brain and stimulation seeded analyses

A whole-brain FBA was performed separately for each hemisphere on the native FOD template to identify associations with lateralised preoperative severity. Associations of preoperative severity lateralised to the maximally affected upper limb were also performed on the flipped FOD template. Analyses were conducted independently for ET and DT groups, as well as for both groups combined.

A stimulation seeded analysis was performed on the native FOD template to identify associations in left and right hemispheres with lateralised postoperative change for ET and DT cohorts separately, as well as both groups combined.

Age and sex were included as covariates for all models. Intracranial volume was additionally included for FC_log_ and FDC metrics (following the same reasoning for combat correction). Disorder was an additional covariate for combined cohort analyses. All variables were demeaned and scaled to unit-variance to circumvent matrix rank-deficiency. Connectivity-based fixel enhancement – a permutation-testing technique at every voxel and direction – was used to control family-wise error rate (*FWE*) by employing threshold-free clustering along each tract (*fixelcfestats*). Non-parametric statistical inference using 5000 random permutations of the data was used. Contrasts were performed for each direction and significant associations were defined according to a threshold of *p* < 0.05*_FWE_*.

For all FBA analyses, we focused specifically on FDC as it has previously been described as the most comprehensive metric describing fixel morphometry [17,50]. Post-hoc exploration of FC_log_ and FD metrics were conducted following statistically significant FDC analyses to assess the specific contributions of each morphometric component.

### Dentato-rubro-thalamic Tract Analyses

The mean FDC value was extracted from each tract for each patient. Multiple linear regressions were performed for the mean tract FDC metric with lateralised preoperative tremor severity and DBS improvement independently and adjusted for age, sex and intracranial volume. False discovery rate (*FDR*) correction was applied to the significance values independently for each group (DT, ET and combined) and significant correlations were considered at a threshold of *p* < 0.05*_FDR_*.

## Results

Two patients were excluded from the study due to inadequate diffusion data quality (as per eddyqc) [51]. The final cohort consisted of 17 patients with DT and 19 patients with ET. Of the 19 patients with ET, nine had a resting upper limb component and one patient had additional resting chin component, resulting in 10 patients fulfilling the diagnostic criteria for ET plus. No significant differences with regards to demographic or clinical variables, stimulation positioning or group-based fixel differences were identified between ET and ET plus groups (see Appendix S1). No significant differences in clinical or demographic variables, or group-based fixel differences were identified for the presence of resting tremor (see Appendix S2).

**Figure 2:**
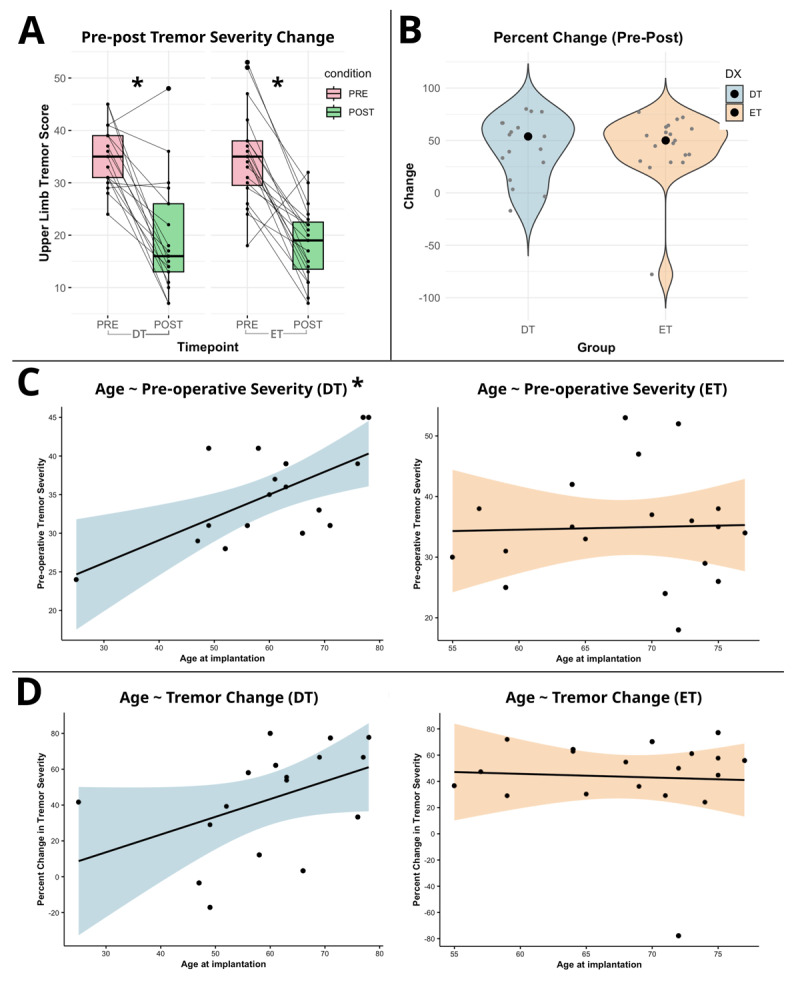
**Clinical and demographic results. A)** Pre-post raw upper limb severity scores for both DT and ET groups. **B)** The postoperative percent change scores of upper limb severity for both DT and ET groups. **C)** Correlations of age and preoperative tremor severity for DT (left) and ET (right). **D)** Correlations of age and postoperative percent change scores of upper limb severity for DT (left) and ET (right). * Indicates statistical significance (*p* < 0.05).

Group demographic and clinical comparisons are summarised in Table 1. Significant reductions of upper limb tremor severity were observed pre-and-post DBS for both patients with ET (*t* = 7.04, *p* = 1.42e-06) and DT (*t* = 5.43, *p* = 5.30e-05). Age at implantation was significantly positively correlated with increased preoperative tremor severity (*R*_p_ = 0.66; *p* = 0.001), and positively but not significantly correlated with DBS improvement (*R*_p_ = 0.38; *p* = 0.096) in patients with DT. Age at implantation was not correlated with preoperative tremor severity (*R*_p_ = –0.03; *p* = 0.870) or DBS improvement (*R*_s_ = –0.01; *p* = 0.946) in patients with ET. Disease duration was not significantly correlated with age (DT: *R*_p_ = 0.28, *p* = 0.260; ET: *R*_p_ = 0.34, *p* = 0.149), preoperative upper limb tremor severity (DT: *R*_p_ = 0.13, *p* = 0.615; ET: *R*_p_ = –0.1, p = 0.665) or postoperative change (DT: *R*_p_ = –0.32, *p* = 0.205; ET: *R*_s_ = –0.10, *p* = 0.662). Main results can be visualised in Figure 2.

**Table 1:** Demographic and clinical information.


	DT	ET/ETp	*p*-VALUE

Age (years)	60.1 ± 13.5; 62	69 ± 6.7; 69.5	0.02*

Disease duration (years)	26.1 ± 11.62; 27	28.1 ± 15.4; 27.5	0.673

Sex (F: *N*; %)	5; 32	7; 39	0.915^†^

Preoperative UL severity (left + right)	35 ± 8.3; 31	35.8 ± 8.4; 35	0.967

Postoperative UL severity (left + right)	19.5 ± 11; 16	18 ± 6.6; 19.5	0.711

Follow-up duration (months)	12.2 ± 3.7; 12	13.1 ± 4.7; 12	0.719

Postoperative change (%)	50.1 ± 35.8; 62.4	55.6 ± 19.1; 56.3	0.782

Stimulation amplitude (left + right; mA) ^a^	3.4 ± 0.6; 3.3	3.4 ± 0.5; 3.3	0.962


*Note*. Values are presented as mean ± standard deviation; median, except sex which is presented as the number, and percentage of females. *^a^* Values reflect only the active electrode for patients presenting with unilateral configuration at follow-up. * Indicates statistical significance. ^†^ Indicates χ^2^ test. Abbreviations: dystonic tremor, DT; essential tremor, ET; female, F; upper limb; UL.

One patient (DT) was excluded from VTA modelling due to corruption of their postoperative CT image. At follow-up, two patients had a unilateral stimulation configuration with one electrode switched off. Electrode implantation positions were deemed satisfactory in all patients (see Figure 2A). Full cohort study parameters are presented in Table S1.

**Figure 3:**
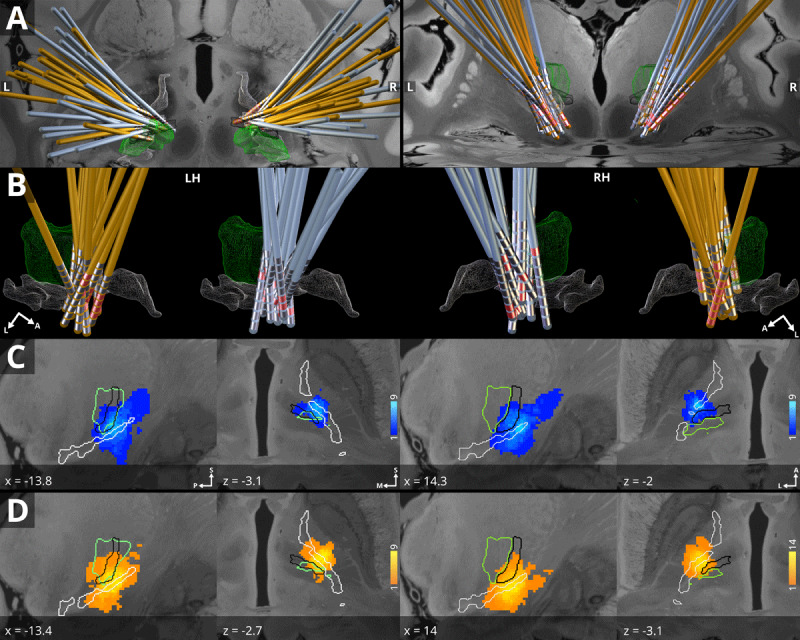
**DT and ET group electrode renderings (A; B) and stimulation volume N-maps (C; D). A)** electrodes are displayed for DT (blue) and ET (orange) groups from axial (left) and coronal views. **B)** Electrodes for each group on the left hemisphere (LH) and right hemisphere (RH). **C)** N-maps are displayed from sagittal (left panels) and axial (right panels) views with corresponding MNI152NLin2009bAsym co-ordinates and panel orientations. Colour bars on the right indicate the number of patient stimulation overlaps for a given voxel. Renderings and outlines show the VIM (green), VOp (black) and zona incerta (white) defined from the DBS Intrinsic Template atlas [52], a manually curated atlas of subcortical structures, superimposed on an ultra-high resolution (100 µm) 7T MRI template in MNI152NLin2009bAsym space [53]. Abbreviations: anterior, A; lateral, L; left hemisphere, LH; medial, M; posterior, P; right hemisphere, RH; superior, S.

Group centroids were predominantly located along the inferior border of VOp and the zona incerta for both groups (Figure 3). For DT, group centroid co-ordinates were located at MNI = –13.8, –12.6, –3.1 (left hemisphere) and MNI = 14.3, –11.4, –2 (right hemisphere). For ET, group centroid co-ordinates were located at MNI = –13.4, –12.9, –2.7 (left hemisphere) and MNI = 14, –11.9, –3.1 (right hemisphere). No significant differences were observed in the variance of stimulation centroid co-ordinates (relative to the group centroid) between ET and DT for the left hemisphere (DT, 4.1 mm; ET, 1.6 mm; *t* = –0.7; *p* = 0.470) or right hemisphere (DT, 2.6 mm; ET, 3.3 mm; *W* = 117; *p* = 0.256).

### Preoperative tremor severity whole-brain analysis

No significant associations of fixel metrics with preoperative tremor severity were identified when each disorder group was analysed alone (for lateralised standard template analyses and flipped laterality analyses). A trend towards significance was observed for the combined group analysis for left lateralised preoperative tremor severity (*p_peak_* = .055). No significant fixels were identified for the right lateralised preoperative tremor severity analysis (*p_peak_* = .132).

**Figure 4:**
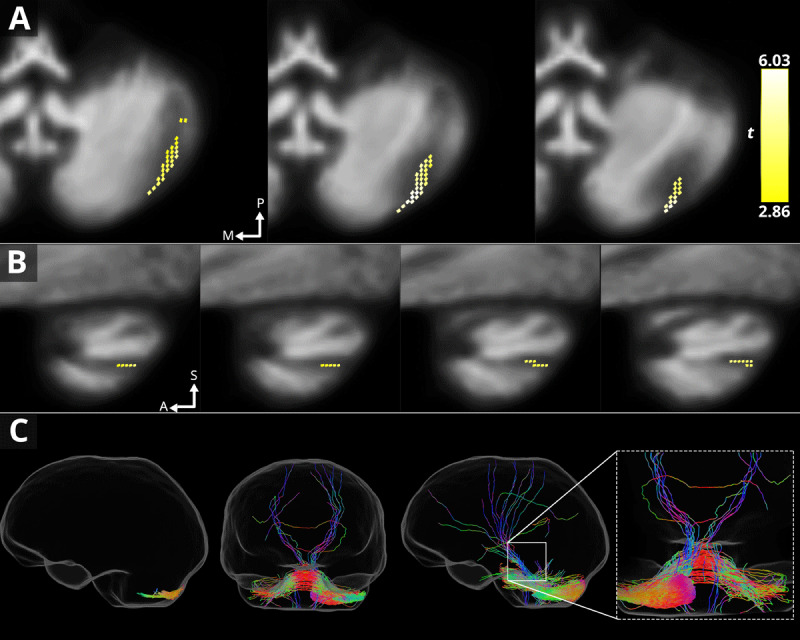
**Fixels with associations of increased FD and FDC changes with increased preoperative tremor severity in patients with essential and dystonic tremor**. Fixels are displayed as consecutive coronal **(A)** and sagittal **(B)** sections. The colour bar indicates the *t*-value for each fixel. From left to right **(C)**: Short range connections (sagittal), long range connections (coronal) and a magnification of non-decussating and decussating cerebello-thalamo-cortical tracts. Abbreviations: anterior, A; lateral, L; medial, M; superior, S.

For the flipped analyses, no significant fixels were identified when each disorder group was analysed alone. When patients were analysed together, a cluster of FDC (*p*_peak_ = .010; 2.86 ≥ *t* ≤ 6.03) was significantly positively associated with preoperative tremor severity, located within white matter adjacent to lobule VII. A cluster of fixels located in the same area were identified when assessing FD (*p*_peak_ = .017; 2.71 ≥ *t* ≤ 5.92) in the whole brain. No significant fixels were identified for FC_log_. Tractography was seeded from identified significant FDC fixels to visualise networks that may be plausibly related to pathological changes. Short (max length = 25) and long range (min length = 25) tracts were generated for illustrative purposes only. These results are presented in Figure 4 and the FD results are presented in Appendix S4.

### Dentato-rubro-thalamic tracts

Reconstructed dentato-rubro-thalamic tracts are presented in Figure S2. Biases in the reconstruction of the decussating and non-decussating tracts were evident in the number of required seeds to generate 2000 streamlines (left non-decussating tract: 184617; right non-decussating tract: 118988; left decussating tract: 1512632; right decussating tract: 1024617).

Uncorrected significant associations were observed for the mean FDC of the right non-decussating dentato-rubro-thalamic tract (*t* = 2.18; *p* = .046) and the left decussating dentato-rubro-thalamic tract (*t* = 2.40; *p* = .030) with postoperative left upper limb change in patients with ET. However, following FDR correction, no significant relationships for any tract with preoperative severity or postoperative change, for DT, ET or when groups were analysed together, were observed (all –1.6 ≥ *t* ≤ 2.4; *p_FDR_* ≥ 0.146).

## Discussion

In the present exploratory study, we performed FBA to assess associations of metrics derived from white matter microstructure in relation to preoperative tremor severity in patients with ET and DT. Furthermore, using modelling from DBS, we attempted to identify associations of metrics derived from white matter microstructure connected to VTA, with postoperative tremor improvements. Finally, we extracted metrics from dentato-rubro-thalamic tracts, comprising a key substrate for therapeutic targeting in DBS, and tested for associations with preoperative tremor severity and postoperative change.

We did not identify differences in stimulation positioning, either in the variance of individual positioning within groups or the differences between groups (based on the centers of gravity). These findings contrast with a previous “sweestpot” modelling study that identified a significantly more anterior positioning of group stimulation in patients with DT relative to ET [54]. Notably, in our cohorts, stimulation volumes were located more inferiorly, overlapping the zona incerta, and required higher stimulation amplitudes to achieve sufficient benefit. This discrepancy may reflect differences in targeting strategy, bilateral versus unilateral implantations, the use of electrodes with a greater span of contact points, and a more extended follow-up period used in the present study.

Although we could not identify disorder-specific white matter microstructural alterations in the present study, our findings suggest a potential nidus underpinning tremor severity across disorders. White matter associations were in the vicinity of lobule crus II which has frequently been implicated in imaging studies related to tremor. For instance, functional MRI studies have noted a negative correlation between the functional connectivity of the left lobule crus II and the left primary motor cortex with tremor severity in ET patients [55]. Furthermore, right lobule crus II was shown to be active during functional recordings of tremor-related activity in patients with DT [7]. Increased effective connectivity of the left lobule crus II with the right supplementary motor area has been observed following focused ultrasound thalamotomy [56]. Finally, structural MRI evidence has identified decreased grey matter volume in the left lobule crus II in patients with ET compared to healthy controls [57].

An increase in FD, and FDC may be taken as evidence for an increase of intra-axonal volume within the region [17]. Enhanced FD, particularly in combination with cross-sectional change, is theorised to reflect an increased ability to propagate activity. Importantly, it has been suggested that ET and DT may not reflect disorders of white matter per se, but instead, may be characterised by the overuse of specific pathways [10], which is in line with reported pathological hyperexcitation in ET and DT [10,58]. Given that dentato-rubro-thalamo-motor tracts were observed as tracts connected to the affected area, pathological tremulous activity may proliferate from such regions of the cerebellum.

Alternatively, increased FDC may reflect histopathological changes in the cerebellum, that have predominantly been reported for ET. Such changes include GABAergic basket cell hypertrophy [59], and swelling of Purkinje cell axons, known as torpedos [60]. Torpedos have also been noted in dystonia, but to a lesser degree than ET [60,61].

The undertaking of a combined group analysis was motivated by findings from a study which identified similarly abnormally altered imaging markers in patients with ET or DT relative to healthy controls [10]. Elevated GABA+ in the cerebellum using magnetic resonance spectroscopy, as well as reduced mean diffusivity in the cerebellum and enhanced fractional anisotropy in the corticospinal tract and red nucleus from diffusion tensor metrics were observed in both ET and DT groups, relative to healthy controls. The authors propose such shared deficits to represent phenomenological, and not etiological similarities.

The rationale for combining patients with ET into a single group stem from a lack of current sufficient evidence to confirm the disorders as distinct entities [5,62], with the consideration that ET plus may represent a manifestation of the same disorder along a continuum [63]. Although group sizes were small, we did not identify significant differences between patients with ET and ET plus in clinical/demographic variables, stimulation positioning or microstructural fixel assessment. Interestingly, in a study focused on voxel-based morphometry of the cerebellum, volume differences were not observed when patients with ET were compared to ET plus (with resting tremor), or when either group were compared with healthy controls. When groups were combined and compared with healthy controls, significantly reduced grey matter volume, specifically of crus II, was identified, corroborating the finding in the present study [64]. No associations with tremor severity were evaluated, however.

We did not identify associations for fixel metrics in tracts seeded from stimulation volumes, or in reconstructed dentato-rubro-thalamic pathways. In the absence of catastrophic damage to targeted tracts, where function within the circuitry is preserved, tremor mediation following DBS may depend solely on the accuracy of targeting. Indeed, accumulating evidence indicates a closer distance between the stimulation site to cerebello-thalamic tracts is a crucial mediating factor for therapeutic success in both ET, and DT [12,14,65]. A potential hypothesis that may explain inter-individual variability is that patients with reduced integrity of bundles that mediate tremor may require increased precision of targeting to preserved pathways to achieve satisfactory alleviation. Although, this is purely speculative it may warrant exploration in the future.

## Limitations

The result of combining multiple scanning acquisition schemes, in combination with a relatively small sample size, on study outcomes is not well characterised. Despite this, we implemented various steps to account for variance associated with these potential differences. Furthermore, given that acquisition schemes were relatively well balanced across disorder groups, we do not assume any biases in favour of detecting effects in any given group.

An absence of microstructural white matter changes relating to postoperative DBS changes may be a result of limited diffusion quality, with tracts that mediate tremor improvement not being accurately modelled at the group level. For example, pallidothalamic tracts may better capture pathophysiology in DT [54], but given their tortuous trajectory and minute scale, may not be accurately captured within the data [66]. Furthermore, non-decussating streamlines were more easily reconstructable, despite decussating fibers constituting 2/3 of total cerebello-thalamic fibers. This bias is consistent with previous reports [49], and reflects an inherent limitation of tractography’s inability to accurately resolve scenarios of crossing fibers [67]. Cerebello-thalamic pathways traverse major “bottleneck” areas (invoking partial volume effects), including cortico-pontine projections of the brainstem and thalamo-cortical radiations [68], and as such, false positive fixel inclusion may arise from erroneously included neighbouring bundles.

It may also be postulated that the postoperative time-point is too extensive, and that tremor resolution is primarily due to the plastic changes triggered by DBS. Nonetheless, there exists a need to balance against using a time-point that is too brief, as surgical planning sessions can take months to years to decipher optimal stimulation configurations [69]. Future work assessing microstructural metric changes using higher resolution data at multiple, well-controlled postoperative timepoints may provide greater insight into the utility of preoperative metric projections.

Finally, the nature of aligning DWI based on the highest lateralised severity may call into question the handling of the brain’s natural asymmetry, and the potential asymmetric effects induced by disease and disorder. Nonetheless, we can be confident that flipping did not fundamentally alter the underlying white matter microstructure as trends towards significance for fixels within the same region were observed within the left lateralised analysis. Such a finding indicates that the greater number of left-lateralised patients in the study may be driving such an effect.

## Conclusion

This work provides insight into white matter microstructure that may mediate tremor severity in DT and ET. This study also highlights the need for future work to determine the utility of preoperative white matter microstructure assessment in relation to outcomes following DBS.

## Additional File

The additional file for this article can be found as follows:

10.5334/tohm.904.s1 Supplementary material. Appendix S1 to S4.

## References

[1] Louis ED, McCreary M. How Common is Essential Tremor? Update on the Worldwide Prevalence of Essential Tremor. Tremor Other Hyperkinet Mov (N Y). 2021; 11: 28. DOI: 10.5334/tohm.632 34277141 PMC8269764

[2] Bhatia KP, Bain P, Bajaj N, Elble RJ, Hallett M, Louis ED, et al. Consensus Statement on the classification of tremors. from the task force on tremor of the International Parkinson and Movement Disorder Society. Mov Disord. 2018; 33: 75–87. DOI: 10.1002/mds.27121 29193359 PMC6530552

[3] Espay AJ, Lang AE, Erro R, Merola A, Fasano A, Berardelli A, et al. Essential pitfalls in “essential” tremor. Mov Disord. 2017; 32: 325–31. DOI: 10.1002/mds.26919 28116753 PMC5359065

[4] Pandey S, Bhattad S, Hallett M. The Problem of Questionable Dystonia in the Diagnosis of “Essential Tremor-Plus”. Tremor Other Hyperkinet Mov (N Y). 2020; 10: 27. DOI: 10.5334/tohm.539 32864186 PMC7427675

[5] Rajan R, Anandapadmanabhan R, Vishnoi A, Latorre A, Thirugnanasambandam N, Dipani A, et al. Essential Tremor and Essential Tremor Plus Are Essentially Similar Electrophysiologically. Movement Disord Clin Pract. 2024; 11: 136–42. DOI: 10.1002/mdc3.13941 PMC10883406 38386479

[6] van den Berg KRE, Helmich RC. The Role of the Cerebellum in Tremor - Evidence from Neuroimaging. Tremor Other Hyperkinet Mov (N Y). 2021; 11: 49. DOI: 10.5334/tohm.660 34820148 PMC8603856

[7] Nieuwhof F, Toni I, Dirkx MF, Gallea C, Vidailhet M, Buijink AWG, et al. Cerebello-thalamic activity drives an abnormal motor network into dystonic tremor. Neuroimage Clin. 2022; 33: 102919. DOI: 10.1016/j.nicl.2021.102919 34929584 PMC8688717

[8] Younger E, Ellis EG, Parsons N, Pantano P, Tommasin S, Caeyenberghs K, et al. Mapping Essential Tremor to a Common Brain Network Using Functional Connectivity Analysis. Neurology. 2023; 101: e1483–94. DOI: 10.1212/WNL.0000000000207701 37596042 PMC10585696

[9] Nicoletti V, Cecchi P, Pesaresi I, Frosini D, Cosottini M, Ceravolo R. Cerebello-thalamo-cortical network is intrinsically altered in essential tremor: evidence from a resting state functional MRI study. Sci Rep. 2020; 10: 16661. DOI: 10.1038/s41598-020-73714-9 33028912 PMC7541442

[10] Bédard P, Panyakaew P, Cho H-J, Hallett M, Horovitz SG. Multimodal imaging of essential tremor and dystonic tremor. NeuroImage: Clinical. 2022; 36. DOI: 10.1016/j.nicl.2022.103247 PMC9668651 36451353

[11] Buijink AWG, van Rootselaar A-F, Helmich RC. Connecting tremors – a circuits perspective. Curr Opin Neurol. 2022; 35: 518–24. DOI: 10.1097/WCO.0000000000001071 35788547

[12] Dembek TA, Petry-Schmelzer JN, Reker P, Wirths J, Hamacher S, Steffen J, et al. PSA and VIM DBS efficiency in essential tremor depends on distance to the dentatorubrothalamic tract. Neuroimage Clin. 2020; 26: 102235. DOI: 10.1016/j.nicl.2020.102235 32172171 PMC7076091

[13] Middlebrooks EH, Okromelidze L, Wong JK, Eisinger RS, Burns MR, Jain A, et al. Connectivity correlates to predict essential tremor deep brain stimulation outcome: Evidence for a common treatment pathway. Neuroimage Clin. 2021; 32: 102846. DOI: 10.1016/j.nicl.2021.102846 34624639 PMC8503569

[14] Coenen VA, Sajonz B, Prokop T, Reisert M, Piroth T, Urbach H, et al. The dentato-rubro-thalamic tract as the potential common deep brain stimulation target for tremor of various origin: an observational case series. Acta Neurochir (Wien). 2020; 162: 1053–66. DOI: 10.1007/s00701-020-04248-2 31997069 PMC7156360

[15] Panyakaew P, Cho HJ, Lee SW, Wu T, Hallett M. The Pathophysiology of Dystonic Tremors and Comparison With Essential Tremor. J Neurosci. 2020; 40: 9317–26. DOI: 10.1523/JNEUROSCI.1181-20.2020 33097635 PMC7687063

[16] Bammer R. Basic principles of diffusion-weighted imaging. Eur J Radiol. 2003; 45: 169–84. DOI: 10.1016/s0720-048x(02)00303-0 12595101

[17] Raffelt DA, Tournier J-D, Smith RE, Vaughan DN, Jackson G, Ridgway GR, et al. Investigating white matter fibre density and morphology using fixel-based analysis. Neuroimage. 2017; 144: 58–73. DOI: 10.1016/j.neuroimage.2016.09.029 27639350 PMC5182031

[18] Dhollander T, Clemente A, Singh M, Boonstra F, Civier O, Duque JD, et al. Fixel-based Analysis of Diffusion MRI: Methods, Applications, Challenges and Opportunities. Neuroimage. 2021; 241: 118417. DOI: 10.1016/j.neuroimage.2021.118417 34298083

[19] Jeurissen B, Leemans A, Tournier J-D, Jones DK, Sijbers J. Investigating the prevalence of complex fiber configurations in white matter tissue with diffusion magnetic resonance imaging. Hum Brain Mapp. 2013; 34: 2747–66. DOI: 10.1002/hbm.22099 22611035 PMC6870534

[20] Raffelt DA, Smith RE, Ridgway GR, Tournier J-D, Vaughan DN, Rose S, et al. Connectivity-based fixel enhancement: Whole-brain statistical analysis of diffusion MRI measures in the presence of crossing fibres. Neuroimage. 2015; 117: 40–55. DOI: 10.1016/j.neuroimage.2015.05.039 26004503 PMC4528070

[21] Rau Y-A, Wang S-M, Tournier J-D, Lin S-H, Lu C-S, Weng Y-H, et al. A longitudinal fixel-based analysis of white matter alterations in patients with Parkinson’s disease. Neuroimage Clin. 2019; 24: 102098. DOI: 10.1016/j.nicl.2019.102098 31795054 PMC6889638

[22] Zito GA, Tarrano C, Ouarab S, Jegatheesan P, Ekmen A, Béranger B, et al. Fixel-Based Analysis Reveals Whole-Brain White Matter Abnormalities in Cervical Dystonia. Mov Disord. 2023; 38: 1187–96. DOI: 10.1002/mds.29425 37148555

[23] Oh SL, Chen C-M, Wu Y-R, Valdes Hernandez M, Tsai C-C, Cheng J-S, et al. Fixel-Based Analysis Effectively Identifies White Matter Tract Degeneration in Huntington’s Disease. Front Neurosci. 2021; 15: 711651. DOI: 10.3389/fnins.2021.711651 34588947 PMC8473742

[24] Keller SS, Glenn GR, Weber B, Kreilkamp BAK, Jensen JH, Helpern JA, et al. Preoperative automated fibre quantification predicts postoperative seizure outcome in temporal lobe epilepsy. Brain. 2017; 140: 68–82. DOI: 10.1093/brain/aww280 28031219 PMC5226062

[25] Mosley PE, Paliwal S, Robinson K, Coyne T, Silburn P, Tittgemeyer M, et al. The structural connectivity of subthalamic deep brain stimulation correlates with impulsivity in Parkinson’s disease. Brain. 2020; 143: 2235–54. DOI: 10.1093/brain/awaa148 32568370

[26] Tournier J-D, Smith R, Raffelt D, Tabbara R, Dhollander T, Pietsch M, et al. MRtrix3: A fast, flexible and open software framework for medical image processing and visualisation. Neuroimage. 2019; 202: 116137. DOI: 10.1016/j.neuroimage.2019.116137 31473352

[27] Schilling KG, Blaber J, Huo Y, Newton A, Hansen C, Nath V, et al. Synthesized b0 for diffusion distortion correction (Synb0-DisCo). Magnetic Resonance Imaging. 2019; 64: 62–70. DOI: 10.1016/j.mri.2019.05.008 31075422 PMC6834894

[28] Smith SM, Jenkinson M, Woolrich MW, Beckmann CF, Behrens TEJ, Johansen-Berg H, et al. Advances in functional and structural MR image analysis and implementation as FSL. Neuroimage. 2004; 23 Suppl 1: S208–219. DOI: 10.1016/j.neuroimage.2004.07.051 15501092

[29] Andersson JLR, Sotiropoulos SN. An integrated approach to correction for off-resonance effects and subject movement in diffusion MR imaging. Neuroimage. 2016; 125: 1063–78. DOI: 10.1016/j.neuroimage.2015.10.019 26481672 PMC4692656

[30] Hoopes A, Mora JS, Dalca AV, Fischl B, Hoffmann M. SynthStrip: skull-stripping for any brain image. NeuroImage. 2022; 260: 119474. DOI: 10.1016/j.neuroimage.2022.119474 35842095 PMC9465771

[31] Neudorfer C, Butenko K, Oxenford S, Rajamani N, Achtzehn J, Goede L, et al. Lead-DBS v3.0: Mapping deep brain stimulation effects to local anatomy and global networks. NeuroImage. 2023; 268: 119862. DOI: 10.1016/j.neuroimage.2023.119862 36610682 PMC10144063

[32] Friston KJ, editor. Statistical parametric mapping: the analysis of funtional brain images. 1st ed. Amsterdam ; Boston: Elsevier/Academic Press; 2007.

[33] Avants B, Epstein C, Grossman M, Gee J. Symmetric diffeomorphic image registration with cross-correlation: Evaluating automated labeling of elderly and neurodegenerative brain. Medical Image Analysis. 2008; 12: 26–41. DOI: 10.1016/j.media.2007.06.004 17659998 PMC2276735

[34] Fonov VS, Evans AC, McKinstry RC, Almli CR, Collins D. Unbiased nonlinear average age-appropriate brain templates from birth to adulthood. NeuroImage. 2009; S102. DOI: 10.1016/S1053-8119(09)70884-5

[35] Avants BB, Epstein CL, Grossman M, Gee JC. Symmetric diffeomorphic image registration with cross-correlation: evaluating automated labeling of elderly and neurodegenerative brain. Med Image Anal. 2008; 12: 26–41. DOI: 10.1016/j.media.2007.06.004 17659998 PMC2276735

[36] Schönecker T, Kupsch A, Kühn AA, Schneider G-H, Hoffmann K-T. Automated optimization of subcortical cerebral MR imaging-atlas coregistration for improved postoperative electrode localization in deep brain stimulation. AJNR Am J Neuroradiol. 2009; 30: 1914–21. DOI: 10.3174/ajnr.A1741 19713324 PMC7051288

[37] Horn A, Kühn AA. Lead-DBS: A toolbox for deep brain stimulation electrode localizations and visualizations. NeuroImage. 2015; 107: 127–35. DOI: 10.1016/j.neuroimage.2014.12.002 25498389

[38] Oxenford S, Ríos AS, Hollunder B, Neudorfer C, Boutet A, Elias GJB, et al. WarpDrive: Improving spatial normalization using manual refinements. Medical Image Analysis. 2024; 91: 103041. DOI: 10.1016/j.media.2023.103041 38007978 PMC10842752

[39] Husch A, Petersen MV, Gemmar P, Goncalves J, Hertel F. PaCER – A fully automated method for electrode trajectory and contact reconstruction in deep brain stimulation. NeuroImage: Clinical. 2018; 17: 80–9. DOI: 10.1016/j.nicl.2017.10.004 29062684 PMC5645007

[40] Horn A, Reich M, Vorwerk J, Li N, Wenzel G, Fang Q, et al. Connectivity Predicts deep brain stimulation outcome in Parkinson disease. Ann Neurol. 2017; 82: 67–78. DOI: 10.1002/ana.24974 28586141 PMC5880678

[41] Dhollander T, Mito R, Raffelt D, Connelly A. Improved white matter response function estimation for 3-tissue constrained spherical deconvolution. Proc. Intl. Soc. Mag. Reson. Med, vol. 555, 2019.

[42] Dhollander T, Connelly A. A novel iterative approach to reap the benefits of multi-tissue CSD from just single-shell (+b=0) diffusion MRI data; 2016.

[43] Tournier JD, Calamante F, Connelly A, others. Improved probabilistic streamlines tractography by 2nd order integration over fibre orientation distributions. Proceedings of the international society for magnetic resonance in medicine, vol. 1670, John Wiley & Sons, Inc New Jersey, NJ; 2010.

[44] Smith RE, Tournier J-D, Calamante F, Connelly A. SIFT: Spherical-deconvolution informed filtering of tractograms. Neuroimage. 2013; 67: 298–312. DOI: 10.1016/j.neuroimage.2012.11.049 23238430

[45] Fortin J-P, Parker D, Tunç B, Watanabe T, Elliott MA, Ruparel K, et al. Harmonization of multi-site diffusion tensor imaging data. Neuroimage. 2017; 161: 149–70. DOI: 10.1016/j.neuroimage.2017.08.047 28826946 PMC5736019

[46] Andrews L, Keller SS, Osman-Farah J, Macerollo A. A structural magnetic resonance imaging review of clinical motor outcomes from deep brain stimulation in movement disorders. Brain Communications. 2023; 5: fcad171. DOI: 10.1093/braincomms/fcad171 37304793 PMC10257440

[47] Smith R, Dhollander T, Connelly A. On the regression of intracranial volume in Fixel-Based Analysis; 2019.

[48] Astrom M, Diczfalusy E, Martens H, Wardell K. Relationship between Neural Activation and Electric Field Distribution during Deep Brain Stimulation. IEEE Trans Biomed Eng. 2015; 62: 664–72. DOI: 10.1109/TBME.2014.2363494 25350910

[49] Petersen KJ, Reid JA, Chakravorti S, Juttukonda MR, Franco G, Trujillo P, et al. Structural and functional connectivity of the nondecussating dentato-rubro-thalamic tract. Neuroimage. 2018; 176: 364–71. DOI: 10.1016/j.neuroimage.2018.04.074 29733955 PMC6002752

[50] Petersen M, Frey BM, Mayer C, Kühn S, Gallinat J, Hanning U, et al. Fixel based analysis of white matter alterations in early stage cerebral small vessel disease. Sci Rep. 2022; 12: 1581. DOI: 10.1038/s41598-022-05665-2 35091684 PMC8799636

[51] Bastiani M, Cottaar M, Fitzgibbon SP, Suri S, Alfaro-Almagro F, Sotiropoulos SN, et al. Automated quality control for within and between studies diffusion MRI data using a non-parametric framework for movement and distortion correction. Neuroimage. 2019; 184: 801–12. DOI: 10.1016/j.neuroimage.2018.09.073 30267859 PMC6264528

[52] Ewert S, Plettig P, Li N, Chakravarty MM, Collins DL, Herrington TM, et al. Toward defining deep brain stimulation targets in MNI space: A subcortical atlas based on multimodal MRI, histology and structural connectivity. Neuroimage. 2018; 170: 271–82. DOI: 10.1016/j.neuroimage.2017.05.015 28536045

[53] Edlow BL, Mareyam A, Horn A, Polimeni JR, Witzel T, Tisdall MD, et al. 7 Tesla MRI of the ex vivo human brain at 100 micron resolution. Sci Data. 2019; 6: 244. DOI: 10.1038/s41597-019-0254-8 31666530 PMC6821740

[54] Tsuboi T, Wong JK, Eisinger RS, Okromelidze L, Burns MR, Ramirez-Zamora A, et al. Comparative connectivity correlates of dystonic and essential tremor deep brain stimulation. Brain. 2021; 144: 1774–86. DOI: 10.1093/brain/awab074 33889943

[55] Buijink AWG, van der Stouwe AMM, Broersma M, Sharifi S, Groot PFC, Speelman JD, et al. Motor network disruption in essential tremor: a functional and effective connectivity study. Brain. 2015; 138: 2934–47. DOI: 10.1093/brain/awv225 26248468

[56] Lueckel JM, Upadhyay N, Purrer V, Maurer A, Borger V, Radbruch A, et al. Whole-brain network transitions within the framework of ignition and transfer entropy following VIM-MRgFUS in essential tremor patients. Brain Stimul. 2023; 16: 879–88. DOI: 10.1016/j.brs.2023.05.006 37230462

[57] Ågren R, Awad A, Blomstedt P, Fytagoridis A. Voxel-Based Morphometry of Cerebellar Lobules in Essential Tremor. Front Aging Neurosci. 2021; 13: 667854. DOI: 10.3389/fnagi.2021.667854 34177554 PMC8222624

[58] Simonyan K, Cho H, Hamzehei Sichani A, Rubien-Thomas E, Hallett M. The direct basal ganglia pathway is hyperfunctional in focal dystonia. Brain. 2017; 140: 3179–90. DOI: 10.1093/brain/awx263 29087445 PMC5841143

[59] Lee PJ, Kerridge CA, Chatterjee D, Koeppen AH, Faust PL, Louis ED. A Quantitative Study of Empty Baskets in Essential Tremor and Other Motor Neurodegenerative Diseases. J Neuropathol Exp Neurol. 2019; 78: 113–22. DOI: 10.1093/jnen/nly114 30590599 PMC6330169

[60] Louis ED, Martuscello RT, Gionco JT, Hartstone WG, Musacchio JB, Portenti M, et al. Histopathology of the cerebellar cortex in essential tremor and other neurodegenerative motor disorders: comparative analysis of 320 brains. Acta Neuropathol. 2023; 145: 265–83. DOI: 10.1007/s00401-022-02535-z 36607423 PMC10461794

[61] Prudente CN, Pardo CA, Xiao J, Hanfelt J, Hess EJ, Ledoux MS, et al. Neuropathology of cervical dystonia. Exp Neurol. 2013; 241: 95–104. DOI: 10.1016/j.expneurol.2012.11.019 23195594 PMC3570661

[62] Gionco JT, Hartstone WG, Martuscello RT, Kuo S-H, Faust PL, Louis ED. Essential Tremor versus “ET-plus”: A Detailed Postmortem Study of Cerebellar Pathology. Cerebellum. 2021; 20: 904–12. DOI: 10.1007/s12311-021-01263-6 33768479 PMC8972074

[63] Iglesias-Hernandez D, Delgado N, McGurn M, Huey ED, Cosentino S, Louis ED. “ET Plus”: Instability of the Diagnosis During Prospective Longitudinal Follow-up of Essential Tremor Cases. Front Neurol. 2021; 12: 782694. DOI: 10.3389/fneur.2021.782694 34975736 PMC8716461

[64] Sarica A, Quattrone A, Crasà M, Nisticò R, Vaccaro MG, Bianco MG, et al. Cerebellar voxel-based morphometry in essential tremor. J Neurol. 2022; 269: 6029–35. DOI: 10.1007/s00415-022-11291-9 35852601

[65] Prent N, Potters WV, Boon LI, Caan MWA, de Bie RMA, van den Munckhof P, et al. Distance to white matter tracts is associated with deep brain stimulation motor outcome in Parkinson’s disease. J Neurosurg. 2019: 1–10. DOI: 10.3171/2019.5.JNS1952 31349226

[66] Petersen MV, McIntyre CC. Comparison of Anatomical Pathway Models with Tractography Estimates of the Pallidothalamic, Cerebellothalamic, and Corticospinal Tracts. Brain Connect. 2023; 13: 237–46. DOI: 10.1089/brain.2022.0068. 36772800 PMC10178936

[67] Schilling K, Gao Y, Janve V, Stepniewska I, Landman BA, Anderson AW. Can increased spatial resolution solve the crossing fiber problem for diffusion MRI? NMR in Biomedicine. 2017; 30: e3787. DOI: 10.1002/nbm.3787 PMC5685916 28915311

[68] Schilling KG, Tax CMW, Rheault F, Landman BA, Anderson AW, Descoteaux M, et al. Prevalence of white matter pathways coming into a single white matter voxel orientation: The bottleneck issue in tractography. Hum Brain Mapp. 2022; 43: 1196–213. DOI: 10.1002/hbm.25697 34921473 PMC8837578

[69] Rodríguez Cruz PM, Vargas A, Fernández-Carballal C, Garbizu J, De La Casa-Fages B, Grandas F. Long-term Thalamic Deep Brain Stimulation for Essential Tremor: Clinical Outcome and Stimulation Parameters. Mov Disord Clin Pract. 2016; 3: 567–72. DOI: 10.1002/mdc3.12337 30363558 PMC6178759

