# Supplementary material for: Exploring White Matter Microstructure with Symptom Severity and Outcomes Following Deep Brain Stimulation in Tremor Syndromes
Source: Tremor Other Hyperkinet Mov (N Y). 2024 Aug 28;14:43. doi: 10.5334/tohm.904 (PMC11363889; doi:10.5334/tohm.904)
Supplement: Supplementary material. — Appendix S1 to S4. [file tohm-14-1-904-s1.pdf]

Supplementary material

Appendix S1

Table 1.

Essential tremor and essential tremor plus group comparisons

	**ET (n = 9)**	**ET plus (n = 10)**	***p*-value**
Age	67.4 ± 6.1; 69	68.7 ± 7.5; 71.5	0.698
Disease duration	25 ± 13; 25	32.3 ± 17.1; 30	0.308
Sex (F: N; %)	3; 33.3	4; 40	0.860*
Pre-operative UL severity (left + right)	37 ± 12; 35	33 ± 5.4; 33.5	0.379
Normalised change (%)	35.3 ± 42; 47.5	51.3 ± 16.6; 56.2	0.308

Note. Values are presented as mean ± standard deviation; median, except sex which is presented as number; percentage. * Indicates χ^2 test. Abbreviations: essential tremor, ET; female, F; upper limb, UL

No significant differences in demographic or clinical information were observed when comparing patients with ET to patients with ET plus.

Stimulation differences

Group centroids for ET were located at MNI = -13.6, -12.7, -1.9 (left hemisphere) and MNI = 14, -12.5, -3.6 (right hemisphere). Groups centroids for ET plus were located at MNI = -13.2, -13.2, -3.3 (left hemisphere) and MNI = 14.1, -11.5, -2.8 (right hemisphere). Group N-maps can be visualised in Figure S1.

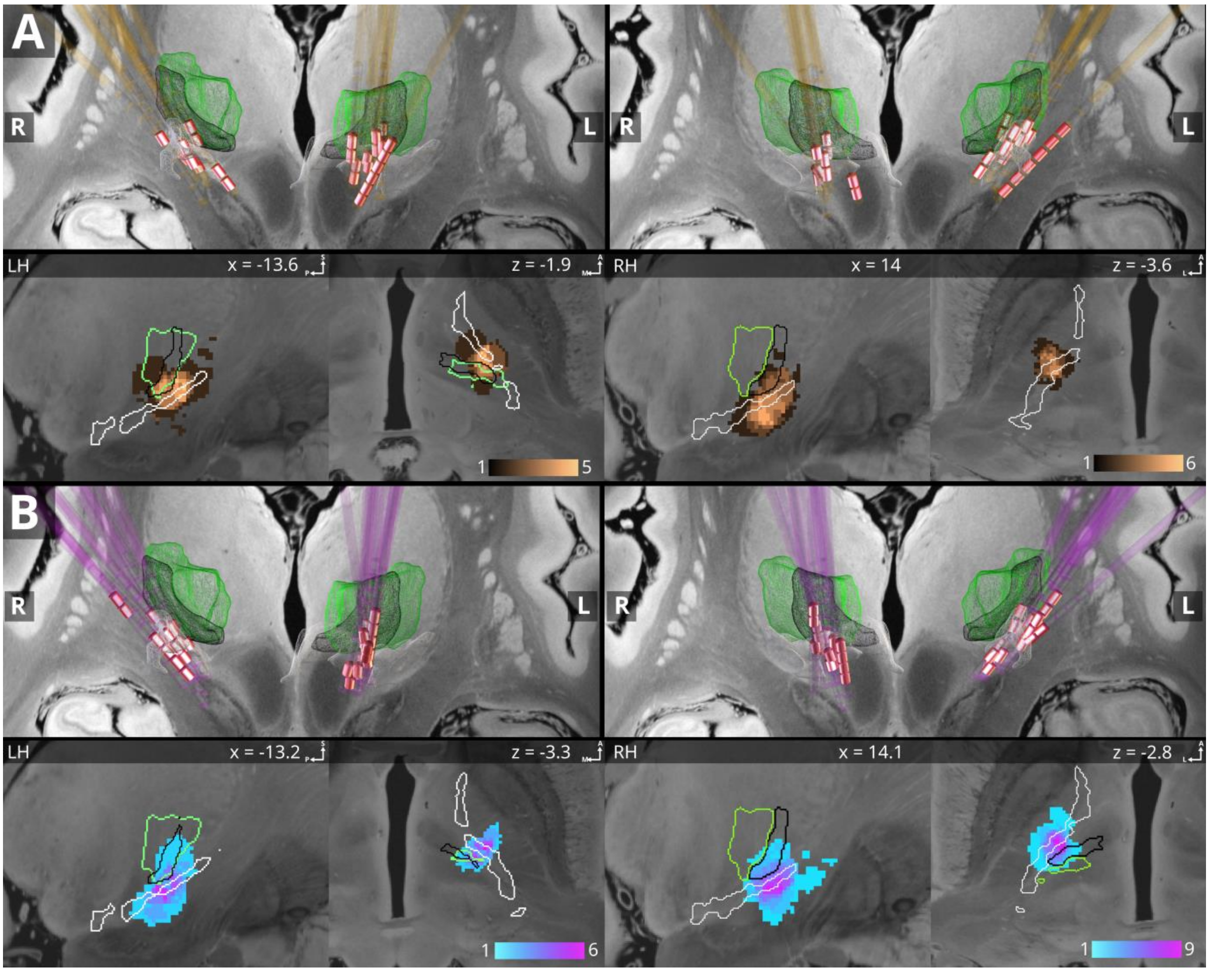

Figure S1. ET and ET plus group N-maps (A; B) and centroid positions (C; D).
Co-ordinates are displayed in MNI152NLin2009bAsym space. Outlines in panels A and B are the VIM (green), VOp (black) and zona incerta (white) defined from the DISTAL) atlas, [Edlow et al, 2018] superimposed on an ultra-high resolution (100µm) 7T MRI template in MNI152NLin2009bAsym space. [Ewert et al, 2019]

No significant differences were identified when comparing the variance of right (t = -0.66; p = 0.516) or left (t = -1.06; p = 0.310) hemisphere centroid distances from each group, between patients with ET and ET plus.

Fixel based analyses

A whole-brain FBA was performed to identify potential group differences between patients with ET and ET plus. No significant differences were observed ($p > 0.2_{FWE}$).

Dentato-rubro-thalamic tract analysis

FDC metrics of the decussating and non-decussating dentato-rubro-thalamic tracts did not significantly differ between groups (all $p = 0.749_{FDR}$).

Appendix S2

Resting versus no resting tremor

Table 1.

Essential tremor and essential tremor plus group comparisons

	**Rest tremor (*N* = 20)**	**No rest tremor (*N* = 16)**	***p*-value**
DX (DT: *N;* %)	10; 50	7; 43.75	0.708*
Age	66.2 ± 9.8; 67.5	61.8 ± 12.2; 63.5	0.248
Disease duration	31.3 ± 14.8; 30	24.1 ± 11.8; 26	0.115

Sex (F: *N*; %)	7; 31.8	5; 35.7	0.808*
Pre-operative UL severity (left + right)	34.8 ± 6; 35	35.1 ± 9.6; 35	0.907
Normalised change (%)	44.8 ± 26.9; 54.7	41.9 ± 35.1; 48.7	0.789†

Note. Values are presented as mean ± standard deviation; median, except DX and sex which are presented as number; percentage. * Indicates χ^2 test; † indicates Wilcoxon rank sum test. Abbreviations: dystonic tremor, DT; disorder group, DX; essential tremor, ET; female, F; upper limb, UL

No significant differences in demographic or clinical information were observed when comparing patients with ET and ET plus.

Fixel based analyses

A whole-brain FBA was performed to identify potential group differences in patients with or without resting tremor. No significant differences were identified ($p > 0.4_{FWE}$).

Dentato-rubro-thalamic tract analysis

FDC metrics of the left and right decussating and non-decussating dentato-rubro-thalamic tracts did not significantly differ between groups (all $t < 1.43$; $p > 0.196_{FDR}$).

Appendix S3

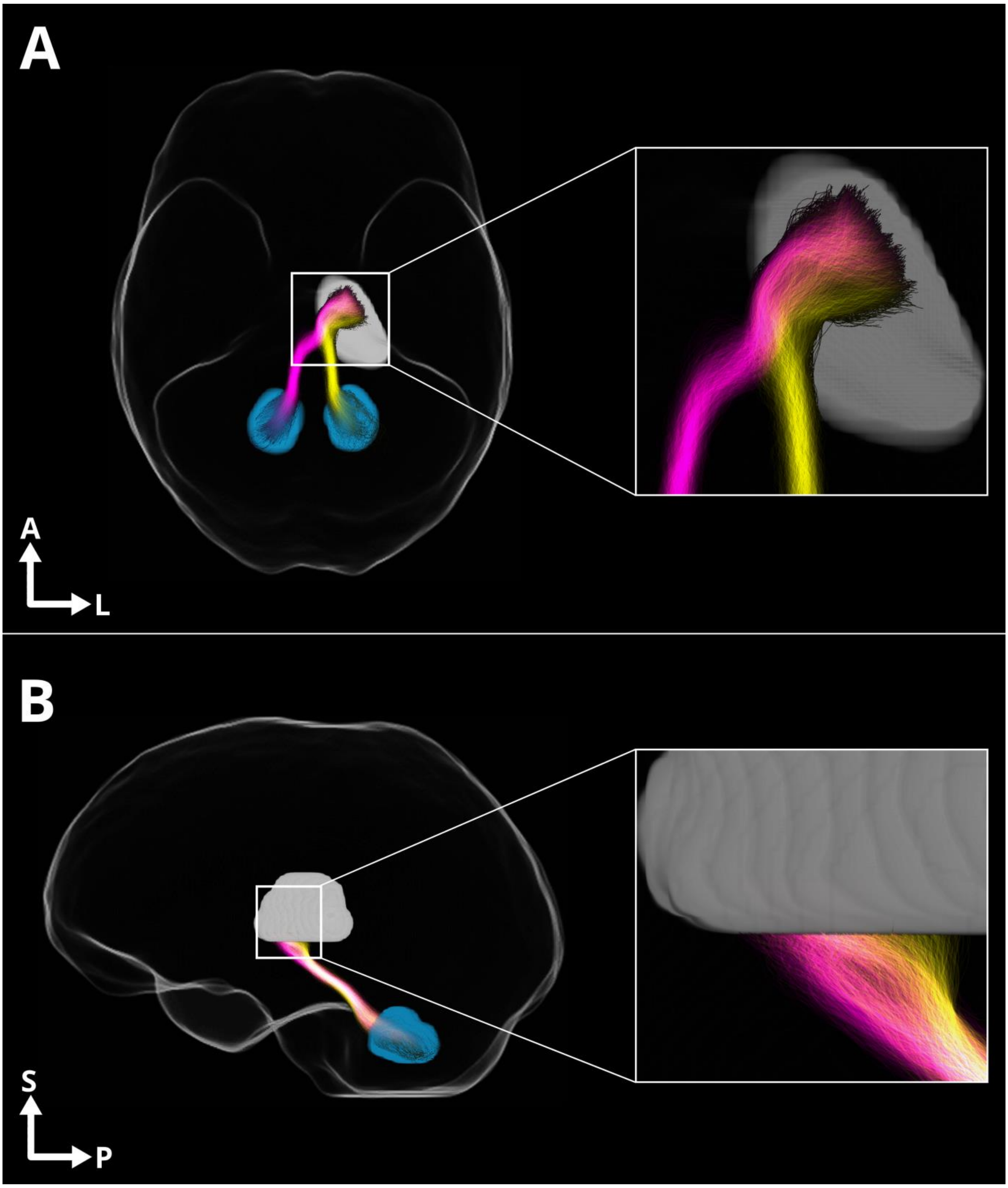

Figure S2. Reconstructed dentato-rubro-thalamic tracts from axial (A) and sagittal (B) views in synopsis with a glass white matter template. Relative to non-decussating streamlines, decussating streamlines showed a more anterior and lateral intersection at the thalamus. Note, tracts projecting to

one hemisphere (left decussating (pink) and right non-decussating (yellow)) are shown for clarity. The thalamus (grey) and dentate nuclei (blue) are displayed as start and end points, respectively. Abbreviations: anterior, A; lateral, L; posterior, P; superior, S

Appendix S4

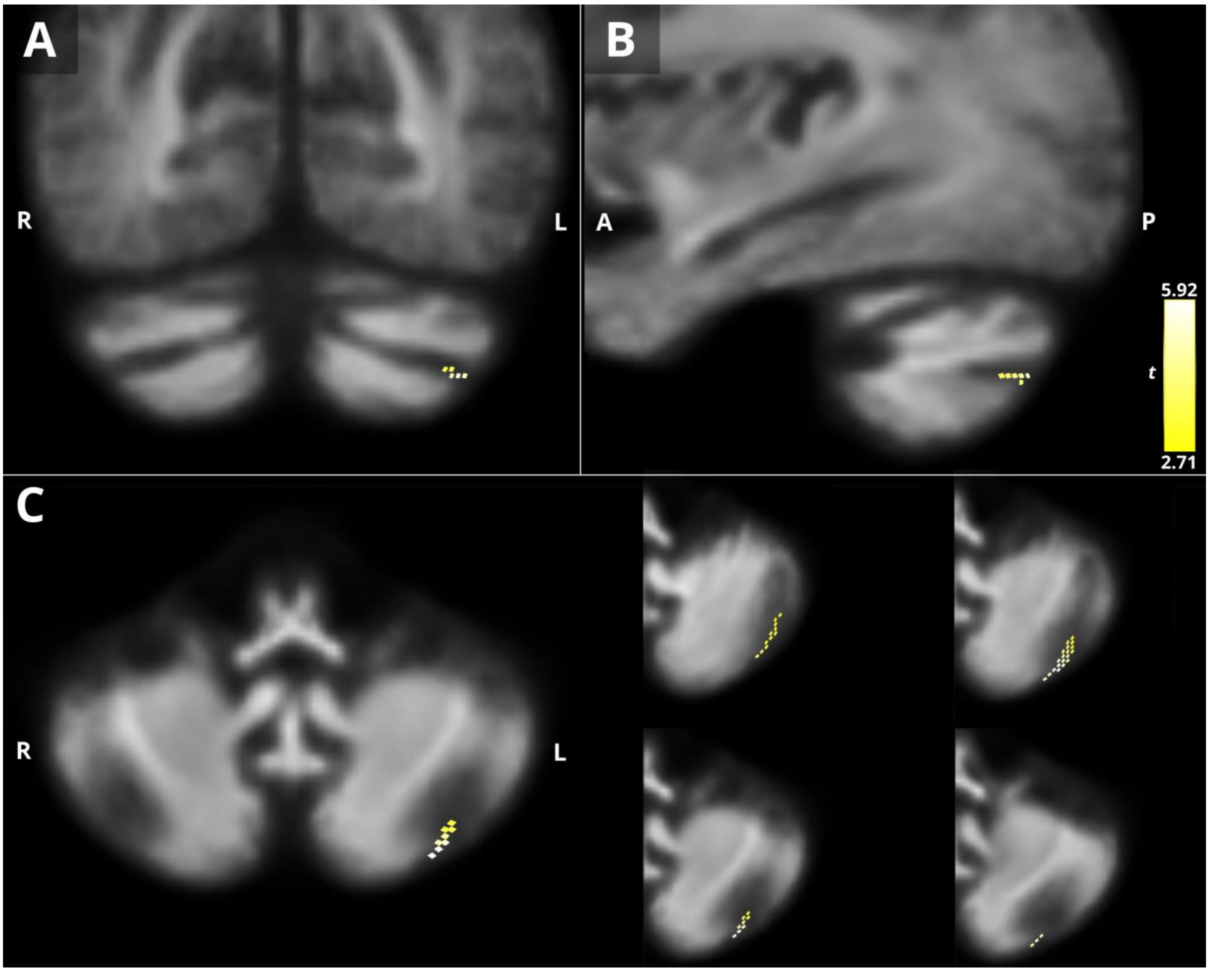

Figure S3. Fixels showing a significant positive association of FD with preoperative tremor severity in DT and ET. Coronal (A), sagittal (B) and axial (C) sections outlining the significant fixels. The colour bar indicates the *t*-value for each fixel. Abbreviations: anterior, A; left, L; posterior, P; right, R

Table S1.

Cohort demographics and stimulation information

Case	DX	Sex	Age	DD	FTMTRS preop. UL severity (left; right)	FTMTRS postop. UL severity (left; right)	Follow-up duration (months)	FTMTRS UL severity % change (left; right)	Stimulation parameters	
									Right	***Left***
22	DT	M	52	38	15; 13	12; 5	12	20; 61.5	3.7mA; C+, K3- (70%), K4- (30%); 179Hz; 50µ	3.9mA; C+, K8- (30%), K9- (70%); 179Hz; 50µ
28	DT	M	58	32	23; 18	21; 15	12	8.7; 16.7	3mA; K0+, K3-; 179Hz; 50µ	2.8mA; C+, K11-; 179Hz; 60µ
30*	DT	M	49	35	24; 17	24; 24	6	0; -41.2	3mA; C+, K1- (50%), K2- (50%); 185Hz; 90µ	3mA; C+, K9-; 185Hz; 90µ
31	ET	M	75	57	19; 19	11; 10	12	42.1; 47.3	3.5mA; C+, K1- (56%), K2- (30%), K3- (14%); 185Hz; 70µ	3.8mA; C+, K9- (37%), K10- (44%), K11- (19%); 185Hz; 50µ
33	ET	M	57	10	20; 18	11; 9	12	45; 50	3.6mA; K0-, K1+; 179Hz; 40µ	3mA; K8-, K9+, 179Hz; 40µ
34	ET	F	75	25	19; 16	4; 4	15	78.9; 75	3.5mA; C+, K1- (33%), K2- (33%),	3.5mA; K10- (33%), K11- (33%), K12-

									K3- (33%); 170Hz; 40µ	(33%), K13+; 170Hz; 40µ
35	DT	M	76	37	24; 15	18; 8	12	25; 46.7	3.7mA; C+, K2- (50%), K3- (50%); 185Hz; 50µ	4.2mA; C+, K8- (50%), K9- (50%); 185Hz; 70µ
38	DT	M	60	23	17; 18	6; 1	16	64.7; 94.4	OFF	4.5mA; C+, K8- (30%), K9- (70%); 179Hz; 80µ
40	DT	M	78	48	21; 24	5; 5	18	76.2; 79.2	2.7mA; C+, K1-; 179Hz; 60µ	2mA; C+, K8-; 179Hz; 60µ
44	DT	M	66	48	14; 16	13; 16	12	7.1; 0	2.5mA; K4-, K5+; 179Hz; 60µ	3mA; K9-, K10+; 179Hz; 60µ
45	DT	M	47	27	13; 16	14; 16	12	-7.7; 0	3.7mA; C+, K4- (12%), K5- (80%), K6- (8%); 170Hz; 50µ	4mA; C+, K13- (90%), K14- (10%), 170Hz; 50µ
46	DT	M	49	33	18; 13	14; 8	12	22.2; 38.5	4.5mA; C+, K1- (70%), K2- (30%); 185H; 80µ	4.5mA; C+, K8- (65%), K9- (35%); 185Hz; 90µ
47	DT	F	71	27	15; 16	3; 4	12	80; 75	3mA; C+, K2- (50%), K3- (50%); 185Hz;	4.2mA; C+, K9- (50%), K10- (50%); 185Hz;

									50µ	60µ
48	ET	M	55	20	16; 14	13; 6	12	18.8; 57.1	4.5mA; C+, K6- (50%), K7- (50%); 179Hz, 40µ	3mA; C+, K8-; 179Hz; 60µ
49	ET	F	71	41	13; 11	11; 6	12	15.4; 45.5	3.5mA; C+, K4; 185Hz; 50µ	3mA; C+, K11-; 185Hz, 60µ
50	DT	M	63	14	21; 18	9; 9	12	57.1; 50	4.5mA; C+, K0- (25%), K1- (25%), K2- (25%), K3- (25%); 185Hz; 50µ	4mA; C+, K8- (25%), K9- (25%), K10- (25%), K11- (25%); 185Hz, 60µ
51	ET	M	72	42	7; 11	9; 23	6	-28.6; -109	3.5mA; C+, K0-; 139Hz; 60µ	2.7mA; K8+, K9-, K10-, K11-, K12-, K13-; 179Hz; 40µ
52	DT	M	25	5	12; 12	8; 6	6	33.3; 50	4.2mA; C+, K5-; 179Hz; 60µ	2.5mA; C+, K15-; 179Hz; 60µ
53	ET	F	59	31	15; 10	5; 2	6	66.7; 80	3.8mA; C+, K2-; 179Hz; 30µ	2.2mA; C+, K9-; 179Hz; 60µ
54	ET	M	64	30	17; 18	9; 4	12	47; 77.8	OFF	4mA; C+, K9-; 179Hz; 40µ
56	DT	F	56	30	17; 14	3; 10	12	82.4; 28.6	3.8mA; C+, K1-; 179Hz; 60µ	2mA; C+, K9-; 179Hz; 60µ

57	ET	F	74	18	17; 12	14; 8	6	17.6; 33.3	4mA; C+, K1-(50%), K2-(50%); 185Hz; 60µ	4.5mA; C+, K10-(50%), K11-(50%); 185Hz; 50µ
58	ET	M	77	55	17; 17	9; 6	12	47.1; 64.7	3.2mA; C+, K3-(50%), K4-(50%); 179Hz; 60µ	3.1mA; C+, K11-(50%), K12-(50%); 179Hz; 50µ
59	DT	M	77	13	21; 24	10; 5	12	52.4; 79.2	3.5mA; C+, K3-; 185Hz; 60µ	3.8mA; C+, K9-, 185Hz; 60µ
60	ET	M	73	15	15; 21	6; 8	12	60; 61.9	3.6mA; C+, K3-, 170Hz; 50µ	3.9mA; C+, K9-, 170Hz, 50µ
61	DT	F	61	25	19; 18	9; 5	22	52.6; 72.2	2.5mA; C+, K2-; 170Hz; 60µ	3mA; C+, K10-; 170Hz; 80µ
62	ET	M	75	13	12; 14	7; 4	10	41.7; 71.4	2.2mA; C+, K2-; 179Hz; 60µ	2.4mA; C+, K10-; 179Hz; 60µ
63	DT	F	69	18	19; 14	7; 4	12	63.2; 71.4	1.5mA; C+, K1-; 179Hz, 50µ	4mA; C+, K10-; 179Hz; 50µ
64	ET	F	68	25	25; 28	11; 13	18	56; 53.6	2.4mA; C+, K1-(10%), K2-(90%); 179Hz; 50µ	3mA; C+, K9-(80%), K10-(20%); 179Hz, 50µ
65	ET	M	65	54	15; 18	15; 8	12	0; 55.6	4mA; C+, K1-(25%), K2-(25%),	4.4mA; K10+, K11-; 179Hz; 90µ

									K3- (25%), K4- (25%); 179Hz; 60µ	
66	ET	M	69	32	24; 23	13; 17	12	45.8; 26.1	2.8mA; C+, K1-; 179Hz; 40µ	2.8mA; C+, K8-; 179Hz; 60µ
67	ET	F	72	16	26; 26	14; 12	12	46.2; 53.8	4.5mA; C+, K0- (60%), K1- (40%); 179Hz; 60µ	4mA; C+, K9- (70%), K10- (30%); 179Hz; 60µ
68	ET	M	64	30	16; 26	7; 8	12	56.3; 69.2	2.7mA; C+, K1-; 179Hz; 60µ	3.1mA; C+, K10-; 179Hz, 60µ
69	ET	F	70	30	17; 20	9; 2	12	47.1; 90	4mA; C+, K1-; 179Hz; 40µ	4mA; C+, K9-; 179Hz; 60µ
70	DT	F	63	11	19; 17	9; 7	15	52.6; 58.8	3mA; C+, K3-; 179Hz; 50µ	2.8mA; C+, K11-; 179Hz; 50µ
71	ET	M	59	4	17; 14	12; 10	18	29.4; 28.6	2mA; C+, K1-; 159Hz; 50µ	4.5mA; C+, K11- (70%), K12- (30%); 185Hz; 50µ

Note. Stimulation parameters are presented as stimulation amplitude (mA), frequency (Hz) and pulse width (µ) for electrode case (C) and contacts (K). OFF indicates stimulation was not being used in the respective hemisphere. Abbreviations: diagnosis, DX; dystonic tremor, DT; essential tremor, ET; female, F; male, M. *. Indicates the patient was not included in lead modelling due to a corrupted CT imag
